# Knowledge and practices of people in Bia District, Ghana, with regard to iodine deficiency disorders and intake of iodized salt

**DOI:** 10.1186/0778-7367-70-5

**Published:** 2012-03-23

**Authors:** Christiana Buxton, Benjamin Baguune

**Affiliations:** 1 Department of Science and Mathematics Education (Health Sciences Education Programme), Faculty of Education, University of Cape Coast, Ghana; 2 District Health Directorate, Ghana Health Services, P. O. Box 29, Essam, Western Region, Ghana

**Keywords:** Knowledge, Practices, Iodized salt, Iodine deficiency disorders, Ghana

## Abstract

**Background:**

Despite numerous educational programmes to create awareness about iodized salt and iodine deficiency disorders (IDD), a survey conducted in the Western Region of Ghana in 2007 revealed that the goitre rate stood at 18.8%; and 78.1% of households consumed iodized salt, which is below the goal of the IDD programme in Ghana which aimed at 90% household consumption of iodized salt by the end of 2005 and sustaining the gains by 2011. It was therefore, considered timely to investigate the knowledge levels and the extent of utilization of iodized salt among the people living in Bia District, the District with the lowest intake (77.4%) of iodized salt based on findings of the 2007 survey.

**Methods:**

This was a descriptive cross-sectional study. It was conducted among a total of 280 household members, mainly in charge of meal preparation, who were interviewed using a structured interview guide. A combination of cluster and simple random sampling techniques was used to select the respondents from all the seven sub- districts in Bia District.

**Results:**

The study revealed that 75.6% of households in the district consumed iodized salt (including households described as occasional users of iodized salt), and knowledge of iodized salt was quite high, as 72% of the respondents knew that not every salt contained iodine. In addition, 69.3% indicated that an inadequate intake of iodized salt can lead to the development of goitre. Despite the high awareness level, only 64.6% of respondents indicated that they exclusively used iodized salt for cooking. The main reason given by exclusive users of common salt was that the price of iodized salt is a little higher than that of common salt.

**Conclusions:**

Although majority of the respondents are aware of the importance of iodized salt and iodine deficiency disorders, only 64.6% exclusively used iodized salt, suggesting that respondents' high knowledge levels did not necessarily translate into an increase in the number of households who used iodized salt. Existing laws and policies on universal salt iodization and quality assurance of iodized salt from the production stage to the distribution/selling stage should be enforced.

## Background

Iodine-deficiency disorders (IDDs) are some of the public health problems that confront 118 countries worldwide, and approximately 1.5 billion people are at risk of preventable IDDs [1-3]. The vulnerable groups particularly at risk include pregnant women, infants and children. In some cases, the developing foetus is affected in the womb [3-5]. Iodine is required to synthesize thyroid hormones which control the body's metabolic rate, and its deficiency results in problems such as abortions, stillbirths, congenital abnormalities, cretinism, goitre and impaired mental function, squinting and mutism [6-10].

In Ghana, it is estimated that 120,000 children born each year are at risk of intellectual impairment because of iodine deficiency. Approximately 15,600 (13%) of these babies are severely impaired and are unable to develop properly, which results in an average of 22 million dollars loss in productivity each year in Ghana. Most of these affected children are also held back by reduced intelligence and mental dullness which are enormous negative educational implications of iodine deficiency [11].

It has been recommended that most developing countries battling with IDD can address the problem in a cost effective way by adding iodine to universally consumed products such as common salt, as done in most industrialized countries [12-16].

In Ghana, the first baseline survey on the state of IDDs in 27 districts was conducted between 1991 and 1994, and the findings revealed a varying degree of endemicity ranging from mild to severe Total Goitre Rates (TGR) [17,18]. On the basis of a baseline survey conducted in 1994, it was found out that IDD was serious in 33% of the 110 surveyed districts [19]. In most of the surveys conducted, the method employed to measure the thyroid volume of subjects was thyroid palpation.

A survey conducted in 2001 revealed that the median urinary iodine level was 77 mcg/L with a range of 28 mcg/L to183 mcg/L [20]. Another study conducted among school children revealed that the median urinary iodine concentration was 67.9 ug/L [21]. Similarly, a study conducted in two districts in the Upper East region in 2007 showed that while there was a drop in overall total goitre rates in the two districts, the median urine iodine levels were below the satisfactory threshold of 100 ug/L. The median urine iodine levels were 51.6 ug/L and 62.56 ug/L in Jirapa and Bongo respectively. The study also found out that only 38.5% and 36.3% of households in Jirapa and Bongo districts respectively were using iodized salt adequately [22].

A market survey conducted in the Western Region in 2007 revealed that the level of patronage of iodized salt was 95.7%. However, this figure dropped to 52% in 2010 [23]. In addition, the market survey showed that 58% of salt sold in markets was iodized, yet below 20 ppm, compared with the mandated iodization level of between 25 and 45 ppm [20,23]. Consequently, it was concluded that the National Salt Iodization Committee and the United Nations' target of 90 per cent plus of Universal Salt Iodization has not been achieved.

A re-analysis of the 1994 baseline data conducted in 2007 indicated that 51.8% rather than 33.3% of Ghana's 1,194 districts were afflicted with IDD, deserving public health attention [19]. Another study conducted in 1998 in the northern parts of Ghana revealed that 68.8% of 1061 subjects had goitre. The subjects were examined for goitre by the palpation method, and every tenth subject examined provided urine for urinary iodine determination. The median urinary iodine level for the subjects was 1.6 micrograms/dl. Seventy two percent (72%) of the subjects had urinary iodine level less than 2 micrograms/dl, 24% had urinary iodine levels in the range 2-5 micrograms/dl and the remainder had urine iodine in the range 5-10 micrograms/dl. The researchers suggested that further studies should be conducted to determine the cause(s) of the IDD endemic [24]. The ignorance of people regarding the importance and sources of iodine to the body could be a contributory factor to this public health problem.

Fortification of salt with iodine has been the most widespread, long-term and effective preventive measure against IDDs since 1920 [25]. To improve consumption of iodized salt, the Universal Salt Iodization (USI) programme was launched in Ghana in 1995. However, in Ghana it has been estimated that approximately 50% of households use iodized salt exclusively [26,27]. Surveys conducted by the Ghana Health Service to assess consumption levels of iodized salts in households revealed that, only 49.1%, 41.5%, 74.1% and 50.8% of households in the country consumed iodized salt solely in 2002, 2003, 2005 and 2006 respectively [28]. The survey conducted in 2006 revealed that only 32.4% of household salt samples were adequately iodized [28]. In addition, only 74% of households consumed iodized salt in Ghana as at 2008, below the national target of 90% which was to have been attained by the end of 2005 and sustained by 2011 [29].

The Medium Term Health Strategy for Ghana towards 'Vision 2020' revised in August 2000, still maintained and emphasized that levels of IDDs were high, especially in the northern part of the country and some parts of the Western Region [30]. It has also been indicated that though the IDD control programme is in place, there are doubts with regard to how the general populace especially in rural communities utilize iodized salt [31].

In the Western Region, surveys carried out to assess household utilization of iodized salt showed that, 53.2%, 67.5% and 78.1% of households consumed iodized salt in 2003, 2005 and 2007 respectively. It was also revealed that, 51.7% of households consumed iodized salt in Bia district (formerly Juaboso-Bia) in 2003, which rose to 76.7% and 77.4% in 2005 and 2007 respectively [32]. The last survey conducted in 2007 showed that 78.1% of households consumed iodized salt in the region, with Bia, the district with the lowest reported iodized salt consumption rate in the Western Region, recording 77.4%, (in a range of 77.4% to 80.8%) [32]. Findings of the 2007 survey further revealed that the goitre rate stood at 18.8% which, according to the study, was quite high [32].

Apart from a survey which was conducted in 2007 to assess household utilization of iodized salt, no other survey has been conducted in the district. Hence the need to undertake this survey to provide current information regarding the utilization of iodized salt in Bia District.

The main objectives of the study, then, were to assess the perceptions, knowledge and practices of people in respect of the use of iodized salt, and to ascertain the current consumption rate of iodized salt in the district. The survey also assessed the iodine concentrations of salt consumed in households in the district.

The findings of this study would be useful to District Health Management Teams (DHMT) which plan promotional and educational programmes on the utilization of iodized salt in Ghana, and to other countries grappling with the problem of IDDs, mainly because of the low utilization of iodized salt.

## Methods

A cross-sectional descriptive household survey design was employed for the study to assess the knowledge and practices of respondents regarding the intake of iodized salt and determine the iodine content of salts used by households [2].

### Sample and sampling procedure

Administratively, the Bia District has seven sub-districts with a total population of approximately 171,325 people [32].

A total sample size of 280 households was randomly selected from all the seven sub- districts in the district. Individuals aged 18 years and above in these households who were responsible for preparing meals constituted the subjects for the study.

A combination of cluster and simple random sampling techniques was used to select households to participate in the study. This was deemed appropriate because of the multiple strata (sub- districts, communities and households structure) in the district. Forty respondents from 40 households were randomly selected from each of the seven clearly demarcated administrative sub-districts. From each sub-district, four communities were randomly selected for the study. Ten respondents were interviewed in each of the 28 communities, resulting in a total number of 280 respondents representing 280 households in the district.

### Instrument

A structured questionnaire with both open-ended and closed-ended questions was used as an interview guide by the researchers to collect the data. The questionnaire was developed employing some questions from similar studies conducted in Ethiopia [2], Mongolia [3] and South Africa [7]. The questionnaire was reviewed by the District Director of Health Services and a public health nutritionist and it was deemed appropriate for use in the communities where the study took place. The questionnaire included a section for observing the type of salt used by households and methods of storage, and the determination of iodine levels in samples of salt used by households, employing the rapid testing kits.

### Data collection procedure

Prior to the administration of the questionnaire, the instrument was pre-tested in 20 households in two communities not selected for the actual study in the district. The necessary modifications and corrections were made on the questionnaire before it was finally administered in the study area. Six Disease Control Field Technicians were trained to assist the researchers in collecting the data. Informed consent was obtained from both heads of the households and the respondents before the interview was conducted.

In each household, permission was sought to run tests on samples of the salt used for cooking to determine their iodine levels. The tests were conducted using the rapid testing kits [33,34]. To determine the iodine levels in the salt samples, colour charts on the kit corresponding to values of 0.1-25 PPM, 25.1-50 PPM, 50.1-75 PPM and 75.1-100 PPM were used.

### Data analysis

At the end of the interviews, questionnaires were checked for completeness and internal consistency. The Statistical Package for the Social Sciences (SPSS) programme software (version 15.0) was used for data entry, and descriptive statistics tests were conducted for the items which were summarised by frequencies and percentages.

## Results

### Socio-demographic status of respondents

Socio-demographic information on the study participants is presented in Table 1. As shown in Table 1, the majority 242(86.4%) were females, suggesting that in the Ghanaian setting women are usually responsible for meal preparation. It also suggests that, health education and awareness programmes which seek to promote the consumption of iodized salt should aim at targeting women groups and organizations at the community level.

**Table 1:** Socio-demographic characteristics of households

Variable	n (%)
**Gender**	
Females	242(86.4)
Males	38(13.6)

**Household number**	
2-4	150(53.6)
5-7	101(36.1)
8-10	23(8.2)
10 and above	6(2.1)

**Respondents' Educational Level**	
No formal education	39(13.9)
Basic/Primary school	48(17.1)
Junior High School(JHS)/Middle School	97(34.6)
Senior High School(SHS)	48(17.1)
Vocational/Commercial	14(5.0)
Tertiary education(Polytechnic/University)	34(12.1)

**Respondents' Occupational Status**	
Farmer	140(50.0)
Trader	46(16.4)
Civil servant	42(15.0)
Apprentice(Hairdresser, Seamstress)	22(7.9)
Student	19(6.8)
Unemployed	11(3.9)

### Knowledge of respondents regarding iodized salt and iodine-deficiency disorders

Responses given by the study participants to the knowledge questions are indicated in Table 2. As shown in Table 2, majority (90.4%) of the respondents indicated that they had heard about iodized salt. The radio was the major medium by which respondents were informed about the importance of iodized salt and iodine-deficiency diseases. Nearly a third (32.9%) of the respondents indicated that the intake of iodized salt is important because it cures goitre whereas 31.4% indicated that the intake of iodized salt enables individuals to remain healthy.

**Table 2:** Knowledge and perceptions of household food caterers about iodized salt and iodine-deficiency disorders

Question/Responses	n (%)
**Heard about iodized salt**	
Yes	253(90.4)
No	27(9.6)

**Source of information about iodized salt**	
Radio	101(39.9)
Friends/relatives	68(26.9)
Television	42(16.6)
Health workers	42(16.6)

**Why intake of iodized salt is important?**	
To cure goitre	92(32.9)
To remain healthy	88(31.4)
To prevent iodine-deficiency disorders(IDDs)	65(23.2)
To grow well	26(9.3)
Better than other salt	2(0.7)
Do not know	7(2.5)

**The result of cooking with un-iodized salt**	
Goiter/swollen thyroid glands	194(69.3)
Low blood level	30(10.7)
Stunted growth in children	24(8.6)
Growing lean/thin	22(7.9)
Do not know	5(1.8)
Others	5(1.8)

**Every salt contains iodine**	
Yes	78(28.0)
No	202(72.0)

**Salt obtained from the sea already contains iodine in the right quantities to support human growth and ensure optimal health**	
Yes	116(41.0)
No	164(59.0)

**Iodine deficiency can expose children to mental retardation**	
Yes	162(58.0)
No	118(42.0)

**Iodine deficiency can lead to growth retardation**	
Yes	140(50.0)
No	140(50.0)

**Iodine content reduces when iodized salt is not stored in enclosed containers**	
Yes	159(57.0)
No	121(43.0)

**Taste of iodized salt is different from that of common salt**	
Yes	167(60.0)
No	113(40.0)

Majority of the respondents (69.3%) indicated that when household meals are prepared without iodized salt, the possible outcome may be goitre. Fifty percent (50%) of the respondents did not agree that iodine deficiency can lead to growth retardation, particularly in children. Regarding storage of iodized salt, 121(43%) did not know that iodine is volatile and therefore escapes into the atmosphere when exposed. Majority (60%) of the respondents indicated that the taste of iodized salt is different from that of common salt.

### Respondents' practices regarding the use of iodized salt

**Figure 1:**
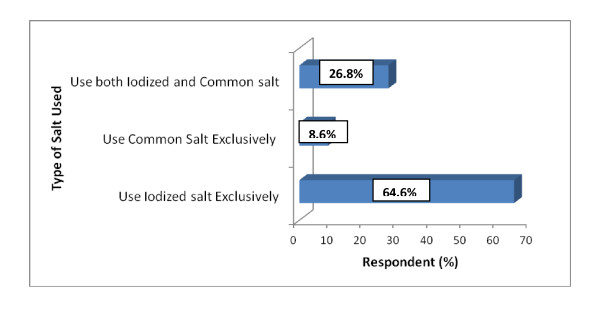
**Use of Iodized Salt by Respondents**.

Figure 1 depicts exclusive users of iodized salt and common salt, and users of both iodized and common salt. The results revealed that majority (64.6%) of the respondents use iodized salt exclusively, whereas 26.8% of the respondents used both common and iodized salt. The respondents who indicated that they used both salts said it was partly due to shortage of iodized salt on the market at certain times. In addition, others indicated that they were unable to distinguish iodized salt from uniodized salt sold on the markets. On the other hand, 8.6% of the respondents reported that they used common salt exclusively, and they attributed this practice to the unavailability of iodized salt on the market. Others indicated that iodized salt was expensive compared with common salt and this influenced their decision to use common salt. For example, at an approximate weight of 500 g, common salt and iodized salt (Annapurna salt) are sold at 35 Ghana pesewas and 70 Ghana pesewas respectively.

Responses given to other questions assessing the practices of respondents are presented in Table 3. The results revealed that majority (51.4%) indicated that they had been using iodized salt for less than five years whereas only 3.6% reported that they had used iodized salt for 16 years and above. The findings show that there has been a remarkable increase in the number of people who consume iodized salt over the past decade.

**Table 3:** Practices of respondents regarding the use of iodized salt

Practices of Respondents (n = 263)	n (%)
**Duration of use of iodized salt**	
Less than 5 years	130(49.5)
From 6-10 years	94(35.7)
From 11-15 years	20(7.6)
16 years and above	9(3.4)
Cannot remember	10(3.8)

**Type/Nature of salt used**	
Coarse	15(5.7)
Granular	13(5.0)
Fine	235(89.3)

**Brand names of salt used by respondents**	
Selfin	10(3.8)
Annapurna	49(18.6)
Unknown	204(77.6)

**Type of container used to store salt at home**	
Container with a lid	165(62.6)
Container without a lid	30(11.5)
Polythene bag	38(14.5)
Rubber sachet	30(11.5)

Salt samples of most (93.9%) of the respondents were tested, whereas salt samples of 6.1% of the respondents were not tested. This was because five out of the 17 did not have salt in their homes at the time of the data collection; and the remaining 12 refused to give a sample of their salt without giving any reasons. An observation made was that, for 75.6% of the respondents whose salt samples were tested, the iodine content was above 25 PPM. The remaining 24.4% of the respondents had salt with iodine content of less than 25 PPM. Out of the 263 salt samples which were tested, the brand names of 77.5% were unknown. This was because in most households, salt is not stored in the original package but is transferred into different storage containers. These findings also suggest that types of salt, particularly with low levels of iodine, are not likely to be readily identified on the market because they do not bear any brand names. Annapurna salt, a common brand of iodized salt in Ghana, was reportedly used by 18.6%, whereas 3.8% used Selfin iodized salt imported from L'Cote de Voire.

Majority (62.6%) of the respondents stored their salt in the recommended way in closed containers. For the 11.5% who stored salt in containers without a lid and another 11.5% who stored it in open rubber sachets, there is the likelihood that the iodine content in the salt might drop because of its volatile characteristic.

### Iodine content in household salt

The majority (75.6%) of the respondents consumed salt with an iodine level of 25 PPM and above, whereas 9.4% and 5.9% consumed salt with an iodine content of less than 25 PPM and 0 PPM respectively, as shown in Table 4. The Ghana Standards Board requires that the iodine content in salt should not be less than 50 PPM at the production point; and it is expected to be at 25 PPM at the retail level. One brand of iodized salt commonly found on the Ghanaian market is Annapurna salt produced by Unilever Ghana limited. Annapurna salt contains an iodine concentration of 50 PPM at the time of packaging and has been endorsed by WHO and ICCIDD.

**Table 4:** Iodine content in household salt samples tested

Iodine Content	**No**.	%
0 PPM	13	5.0
Less than 25 PPM	51	19.4
25 PPM and above	199	75.6

**Total**	**263**	**100.0**

The study participants were asked to suggest measures or strategies to make more people consume iodized salt in the district. The suggestions summarized in Table 5 indicate that a high proportion of the participants (42.3%) thought that the mass media should be actively used to educate the populace about the importance of iodized salt to human health. Others (29.7%) suggested that health workers at the community level should educate people on the importance of consuming iodized salt. This means that public health workers will have to strategize their activities to reach the hard-to-reach parts of the district. Only 2.2% of the respondents suggested that the District Assembly should assist salt distributors to form associations to facilitate effective monitoring to prevent the influx into the market of uniodized salt which is usually cheaper than iodized salt.

**Table 5:** Respondents' views on measures that can be employed to improve on the utilization of iodized salt

Response	Frequency	%
Mass media should actively take part in educating people on importance of iodized salt	119	42.3
Health workers at the community level should educate people on importance of iodized salt	83	29.7
Iodized salt should be distributed freely through health facilities to households	41	14.7
Routine testing for iodine levels in salt sold in the markets and used by households	31	11.1
District Assembly should assist salt distributors to form associations to facilitate the monitoring of salt sold in markets	6	2.2
Total	280	100.0

## Discussion

In Ghana, as in most developing countries, iodization of salt is the major strategy that has been employed to help avert the public health effect of iodine deficiency disorders (IDDs).

Findings regarding the intake of iodized salt in the present study suggest that there has been a remarkable improvement in knowledge about iodized salt compared with the findings of a study conducted by the University of Ghana in collaboration with the Ministry of Health, Ghana, from 1991-1993 on IDDs in Ghana that 98% of the respondents had no knowledge about iodized salt [18]. This improvement has occurred as a result of the Food and Drugs Board amendment Act, Act 523 of 1996 on universal salt iodization and the propagation of health education by the Ministry of Health and the Ghana Health Service creating awareness about the importance of iodized salt. The increase in the knowledge levels over the years suggests that, if awareness creation and educational activities are sustained mainly in the local dialects, it is likely that all Ghanaians would become aware of iodized salt and its importance to human health and wellbeing.

A significant proportion of the respondents (39.9%) indicated that their major source of information about iodized salt was the radio, which could be attributed to the high number of radio stations in the country, especially the three stations in the Bia District. A similar study conducted among rural households also revealed that over 95% of the study participants knew about iodized salt and IDDs mostly through educational programmes broadcast on radios and televisions [3]. Forty two respondents, representing 16.6% indicated that the television and health workers were their main sources of information regarding iodized salt. This low percentage could also be attributed to the fact that majority of the people in Bia are settler farmers who live on their cocoa farms and as such are not likely to have access to public health educational messages propagated through the electronic media or by health workers in the communities. These findings suggest that even though the use of the electronic media is one effective way to improve and sustain peoples' use of iodized salt, settler cocoa farmers, a considerable section of the population in the district, are likely to be left out and may not have access to these educational programmes.

The findings regarding why the intake of iodized salt is important show an improvement over the findings of a similar study conducted by Asibey-Berko (1995) [18] in 30 selected districts from all the ten regions of Ghana within a period of three years (1992-1995) in which it was reported that 1.1% and 0.4% of the respondents indicated that iodized salt prevents goitre and improves the overall health of the individual respectively. It can be inferred that as a result of increased knowledge regarding the importance of using iodized salt and the effects of its deficiency in the diet of an individual, there has also been an increase in the consumption rate of iodized salt as indicated in Figure 1 and Table 3. The knowledge levels of respondents can be described as above average, reflected in the responses of 72% of the respondents who indicated that every salt does not contain iodine. Similarly, the majority of the respondents knew that inadequate intake of iodized salt resulted in the development of goitre. On the other hand, 60% of the respondents indicated that the taste of iodized salt is different from that of common salt, corroborating the report of a similar study conducted in Mongolia, that more than half of the study participants indicated that the taste of iodized salt was not the same as that of common salt. However, in a double-blind study using the same respondents, it was revealed that they could not distinguish the taste differences between iodized and uniodized salt [3]. This misconception regarding the differences between the taste of common salt and iodized salt should be corrected through educational messages, particularly because it can act as a barrier and prevent people from using iodized salt.

Some non-users and occasional users (respondents who used both common salt and iodized salt) reported that common salt was cheaper compared with iodized salt, which influenced their decision in choosing common salt. This finding is supported by a similar study which also reported that the majority of occasional users and non-users of iodized salt indicated that the price of iodized salt was slightly higher than that of common salt [3]. As asserted by Yamada et al. (1998) [3], generally, iodized salt costs more than common salt because of the additional processing cost involved in fortifying common salt with iodine.

A high proportion (57%) of the respondents knew that the iodine content of iodized salt reduces when it is not stored in enclosed containers, by virtue of its volatile characteristic. The findings of a study conducted by Sebotse et al. (2009) [7], indicated that when iodized salt was not stored in closed plastic bags, sealed waterproof materials or closed containers, iodine losses occurred leading to a reduction in the iodine content of the salt before it is consumed.

The finding that 75.6% of respondents consumed iodized salt indicates a slight reduction in the intake of iodized salt compared with the Western Regional Annual Health Sector Report [32] based on that year's household survey which revealed that approximately 77% of the people living in Bia district consumed iodized salt. The present finding suggests that the initial effort by health workers and other collaborators in the dissemination of information to sensitize and create awareness about the importance of consuming iodized salt might be declining. Similarly, it is likely that programmes broadcast through radio stations are also declining. The implication of these findings is that if health education activities are not stepped- up, this downward trend will continue. Other reasons that have been given for the failure of most developing countries to achieve 90% utilization of iodized salt include political factors and logistical problems in the production and distribution of iodized salt [3,35-37].

## Conclusions

The knowledge on iodized salt of people in charge of preparing household meals in the district is relatively high, as most (90.4%) of them knew about iodized salt. However, their knowledge levels were not translated into or reflected in their practices as only 64.6% of households exclusively used iodized salt for cooking and 75.6% of the samples of salt tested had an iodine content of ≥ 25 PPM. A commendable practice of a good proportion (62.6%) of the respondents was the storage of salt in enclosed containers or water proof materials to prevent iodine losses. The research also revealed that, continual and effective use of the electronic media for broadcasting health education programmes in addition to house-to- house visits by public health workers to target populations with no access to the electronic media will help to sustain and improve on the utilization of iodized salt in the district.

### Recommendations and suggestions for further study

On the basis of the findings of the study, recommendations made include the following: In the propagation of educational messages, great effort should be made to correct misconceptions regarding the differences between the tastes of iodized and common salt.

As part of technical surveillance and laboratory services to ensure quality assurance, Environmental Health Workers in the country should be trained by the DHMT on how to conduct iodine tests, and they should be supplied with the rapid test kits to enable them monitor whether salt sold in marketplaces are adequately fortified with iodine, as part of their routine food inspection activities in the district.

Environmental health workers should be empowered by law to prosecute or take legal action against salt sellers found selling salt that is not iodized and also manufacturers who do not comply with the universal salt iodization Act. The Food and Drugs Board should enforce laws that will require all salt produced to bear brand names and contact addresses of manufacturers in order to easily identify uniodized products.

To address the issue of the inability of some consumers to distinguish between iodized salt and uniodized salt, a logo could be inscribed on all recommended brands of iodized salt by the Ghana Standards Board and the Food and Drugs Board to enable consumers, particularly people without any formal education, to identify approved iodized salt brands on the market.

Lastly, regarding the settler cocoa farmers who do not have access to information broadcast through the mass media, perhaps interpersonal communication by disease control officers, public health nurses and other health workers will be a more effective means to disseminate health information to them, as this was reported to be effective in a similar study conducted in Peru [38].

A suggestion for further study is the need to assess the iodine status of school children and pregnant women in particular, which should include the determination of Urinary Iodine Concentration (UIC) levels as recommended by the WHO. This is because these two groups are described as the most vulnerable groups confronted by iodine deficiency disorders.

## Abbreviations

DHD: District health directorate; DHMT: District health management team; GHS: Ghana health service; ICCIDD: International center for the control of iodine deficiency disorders; IDD(s): Iodine deficiency disorders; MDG: Millennium development goal; MUIER: Mean urinary iodine excretion rates; PPM: Parts-per-million; TGR: Total goiter rates; UNICEF: United nations international children's emergency fund/united nations children's fund; USI: Universal salt iodization; WHO: World health organization.

## Competing interests

The authors declare that they have no competing interests.

## Authors' contributions

The authors' responsibilities were as follows: CB supervised the study, participated in the design of the research instrument, reviewed related literature, and participated in discussing findings and making recommendations on the basis of the findings of the study. She finalized the manuscript for submission. BB concieved the idea of this study, participated in the design of the study, and had the major responsibility of coordinating the data collection (interviews and testing of iodine levels of salt) in households, analysis of the data, and presentation of results. He also actively participated in the write-up of the study. Both authors have read and approved the final manuscript.

## Authors' information

CB: Has a background in public health nutrition, has great research interests in micronutrient deficiency problems of public health significance confronting populations particularly in developing countries. She is currently a lecturer in the Health Sciences Education programme in the University of Cape Coast, Ghana. BB: Is a District Disease Control Officer with a background in Health Sciences Education. He currently works at the Ghana Health Service and has actively participated in major projects assessing the health status of populations at both the district, regional and national levels in Ghana.
